# Endoderm Jagged induces liver and pancreas duct lineage in zebrafish

**DOI:** 10.1038/s41467-017-00666-6

**Published:** 2017-10-03

**Authors:** Danhua Zhang, Keith P. Gates, Lindsey Barske, Guangliang Wang, Joseph J. Lancman, Xin-Xin I. Zeng, Megan Groff, Kasper Wang, Michael J. Parsons, J. Gage Crump, P. Duc Si Dong

**Affiliations:** 1 Human Genetics Program, Sanford Burnham Prebys Medical Discovery Institute, 10901 North Torrey Pines Road, La Jolla, CA 92037 USA; 2 Graduate School of Biomedical, Science, 10901 North Torrey Pines Road, La Jolla, CA 92037 USA; 3 Department of Stem Cell Biology and Regenerative Medicine, Keck School of Medicine of University of Southern California, Los Angeles, CA 90033 USA; 4 Department of Surgery, and McKusick-Nathans Institute for Genetic Medicine, Johns Hopkins University School of Medicine, 733N. Broadway, Baltimore, MD 21205 USA

## Abstract

Liver duct paucity is characteristic of children born with Alagille Syndrome (ALGS), a disease associated with *JAGGED1* mutations. Here, we report that zebrafish embryos with compound homozygous mutations in two Notch ligand genes, *jagged1b* (*jag1b*) and *jagged2b* (*jag2b*) exhibit a complete loss of canonical Notch activity and duct cells within the liver and exocrine pancreas, whereas hepatocyte and acinar pancreas development is not affected. Further, animal chimera studies demonstrate that wild-type endoderm cells within the liver and pancreas can rescue Notch activity and duct lineage specification in adjacent cells lacking *jag1b* and *jag2b* expression. We conclude that these two Notch ligands are directly and solely responsible for all duct lineage specification in these organs in zebrafish. Our study uncovers genes required for lineage specification of the intrahepatopancreatic duct cells, challenges the role of duct cells as progenitors, and suggests a genetic mechanism for ALGS ductal paucity.

## Abstract (web summary)

The hepatopancreatic duct cells connect liver hepatocytes and pancreatic acinar cells to the intestine, but the mechanism for their lineage specification is unclear. Here, the authors reveal that Notch ligands Jagged1b and Jagged2b induce duct cell lineage in the liver and pancreas of the zebrafish.

## Introduction

In mammals and zebrafish, the hepatopancreatic ductal system is a network of tubular epithelium connecting hepatocytes of the liver and acinar cells of the pancreas to the intestine. Malformation and dysfunction of hepatopancreatic ducts can lead to pathologies including liver duct paucity and exocrine pancreas insufficiency—as found in patients with Alagille Syndrome (ALGS). ALGS is a congenital disease with a prevalence estimated at 1/70,000 births, based neonatal liver disease^1^. It is associated with heterozygous mutations primarily in *JAG1*, or less frequently in *NOTCH2* ^2–5^. A mechanistic understanding of the neonatal ductal defects in this disease will require rigorous investigation into the function of these genes during hepatopancreas duct development.

The duct lineage, defined by *Sox9* expression, has been proposed as a source of multipotent progenitors that contribute to the development, homeostasis, and regeneration of the liver and pancreas^6^. Subsequent studies on homeostasis and regeneration have both supported and disputed a role for duct cells as source for multipotent progenitors^7–12^. It is generally accepted that during early liver and pancreas development, bipotent (i.e., hepatoblasts) or multipotent cells give rise to both duct and hepatocytes or acinar cells. However, it remains unresolved whether specified duct cells during embryonic development also contribute to acinar and hepatocyte lineages^6, 13–15^. Although Sox9 is considered to be the earliest biliary marker^16^, lineage tracing *Sox9* expression might not be ideal because Sox9 is not exclusively expressed in the duct lineage^8, 17^. Using a more definitive duct lineage tracing CRE line and addressing the functional requirement of liver and pancreas duct cells will be necessary to resolve whether duct cells are a source of multipotent progenitors during organogenesis.

Although specific factors have been implicated in the lineage specification of the endocrine and acinar fates in the pancreas^18, 19^, the genes required for induction of the entire ductal lineages in both the pancreas and liver (intrahepatopancreatic ducts, IHPD) have been elusive. Numerous studies have implicated Notch signaling in the morphogenesis and differentiation of both intrapancreatic ducts (IPDs) and intrahepatic ducts (IHDs)^20, 21^. Ectopic expression of the Notch intracellular domain inhibits expression of hepatocyte and pancreatic acinar genes and enhances duct genes, supporting a role for Notch signaling in duct lineage specification^22, 23^. However, the inability to completely and distinctly block the canonical Notch pathway in the pancreas and liver has confounded efforts to resolve whether this signaling pathway is specifically necessary for duct lineage induction, independent of its recognized requirement for differentiation, expansion, and maintenance of duct cells.

Given functional redundancy among Notch ligands and receptors, the predominant strategy to broadly block canonical Notch signaling has been to manipulate the more general components of the Notch pathway. However, down-regulation of canonical Notch activity by modulating the expression of Notch signaling components did not lead to complete loss of ducts, or yielded contrasting results. For example, while Maml1, Rbpj, Mib1, or Hes1 loss of function in the mouse pancreas can all lead to a reduction in duct lineage markers, the effects on the acinar and endocrine lineages differed^24, 25^. Further, loss of Hes1 and Rbpj also resulted in a broader pancreas hypoplasia phenotype. These differences may be due to varying levels of Notch loss of function or to non-Notch signaling specific effects, because none of the manipulated Notch signaling components are exclusively involved with canonical Notch signaling^26–30^. Furthermore, knockout of Notch receptor genes might also not be ideal because Notch receptors, independent of ligands, have been implicated in ß-catenin signaling^31^.

More direct assessments of the role of Notch signaling in pancreas and liver duct specification may require analyzing the function of Notch ligands. Conditional loss of *Jag1* from the mouse portal vein mesenchyme results in hepatic duct tube morphogenesis defects, leading to the current model suggesting that biliary paucity in ALGS arises via an analogous mechanism–reduced *JAG1* expression from non-endoderm derived cells causes biliary structural, not lineage specification, defects^32, 33^. The potentially incomplete efficiency of Cre/Lox based conditional knock out approaches, combined with the functional redundancy among Notch ligands, make it technically challenging to completely block Notch signaling in the mouse model at the ligand level. In support of redundant roles among Jagged ligands in liver and pancreas development, earlier studies using zebrafish showed that antisense morpholino (MO) knockdown of both *jag1b* and *jag2b* leads to more extensive disruption of pancreas and liver duct development than knockdown of either alone^34^. However, embryos with these factors knocked down continued to express the ductal marker Cytokeratin in what were described as hybrid hepatocytes and acinar cells. Whether early duct cells failed to be induced, appeared initially but failed to properly differentiate, or misdifferentiated into pancreatic endocrine cells, was not determined. Knockdown of either *jag1b* or *jag2b* alone can lead to an increase in the numbers of alpha-cells or beta-cells of the endocrine pancreas^35^. A decreased knockdown effectiveness at later stages is another possibility for the putative ‘hybrid’ cells. Together with concerns regarding off-target effects of morpholino^36^, more reliable loss of function models will be critical to rigorously assess the roles of Jag1b and Jag2b in development and disease.

To investigate the effect of specific, consistent, and complete loss of Jag1b and Jag2b function on liver and pancreas development, we analyzed zebrafish *jag1b* and *jag2b* mutants, using early and late organ cell lineage markers, as well as a transgenic reporter of canonical Notch signaling activity. Our studies yield evidence that Jag1b and Jag2b are the primary ligands required for induction of all detectable canonical Notch signaling and all duct cells within the developing liver and exocrine pancreas and that duct cells are not a significant source of progenitors for the hepatocyte or pancreas acinar lineages. Further, stage specific loss of function studies suggest that, distinct from its later role in preventing pancreatic duct cells from adopting endocrine fate, Notch signaling is required during early pancreogenesis to establish the ductal compartment. Our chimera studies demonstrate that Jagged ligands from endoderm-derived cells within the liver and pancreas are able to directly induce Notch signaling and the duct lineage, a mechanism that contrasts with how Jag1 signaling is thought to regulate duct development in mammals and in ALGS.

## Results

### Liver and exocrine pancreas Notch activity requires Jagged

In zebrafish, both *jag1b* and *jag2b* are widely expressed in the hepatopancreatic domain during development (Supplementary Fig. 1). To assess the role of these ligands using a more consistent and reliable loss of function approach, we examined *jag1b* ^*b1105*^ and *jag2b* ^*hu3425*^ mutants. Both mutants carry nonsense mutations within the N-terminal coding region of their respective genes, and thus are likely to be complete loss-of-function mutants. These mutations do not lead to lethality until juvenile stages, allowing for analysis of Jagged loss-of-function in the developing liver and pancreas. To examine canonical Notch activity in the pancreas and liver, these mutants^37, 38^ were analyzed with a Tp1:GFP transgenic reporter. The Tp1:GFP reporter, which has GFP driven by a regulatory element containing 12 RBPJk binding sites from the promoter of Terminal Protein 1, has been demonstrated to effectively mark cells with canonical Notch signaling activity^39^. With this line, we confirmed that intrapancreatic and intrahepatic ducts, but not the extra hepatopancreatic ducts, have high canonical Notch activity, based on GFP co-expression with AnnexinA4 (Anxa4), which is broadly expressed in fish and mammalian hepatopancreatic ducts^40, 41^. In the exocrine pancreas, all Tp1:GFP expressing cells are also positive for Nkx6.1 (Supplementary Fig. 2a), a transcription factor also expressed in the ductal compartment of the mouse pancreas^42^. Within the developing liver, all cells expressing Prox1 (a broad marker of liver and exocrine pancreas endoderm), that lack the hepatocyte early marker Hnf4a^43^, are Tp1:GFP positive (Supplementary Fig. 2b), indicating that all endoderm-derived cells are either Hnf4a positive or Tp1:GFP positive. These findings demonstrate that within the liver and exocrine pancreas, Notch activity, based on the Tp1 promoter, is strictly limited to and labels all duct cells. Tp1:GFP positive cells that are negative for Prox1 can also be found in and around the liver and pancreas, indicating the presence of non-endoderm, Notch active tissues. Examination of Tp1:GFP expression in conjunction with an endothelial reporter *kdrl:mCherry* ^44^, demonstrates that these Notch active cells are indeed endothelial cells (Supplementary Fig. 2c, d). Tp1:GFP expression is also observed in a subset of pancreatic Islet1 expressing endocrine cells (Supplementary Fig. 2d).

**Fig. 1:**
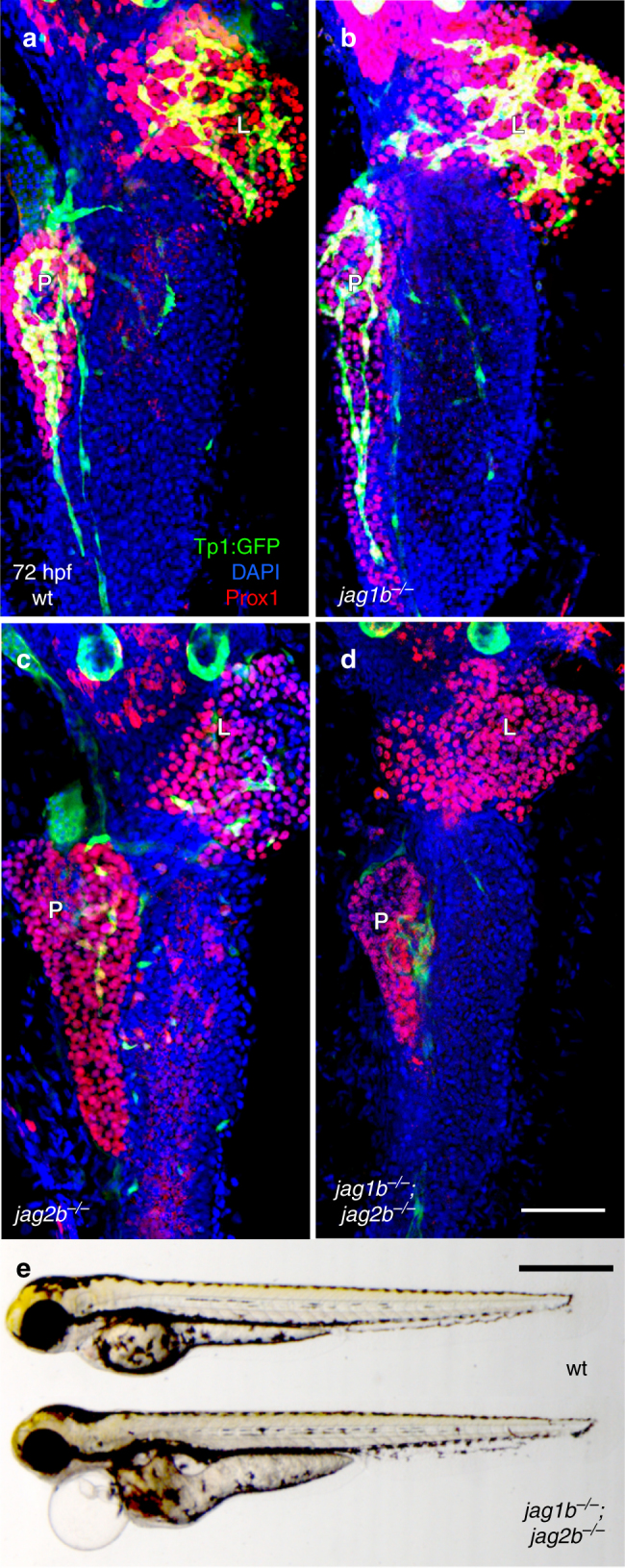
Jag1b and Jag2b are required for all canonical Notch signaling in the developing liver and exocrine pancreas. **a**–**d** 3D rendering of the foregut endoderm of 72 hpf embryos in the canonical Notch signaling transgenic reporter tg(Tp1:GFP) (*green*) background, stained with hepatopancreatic endoderm marker Prox1 antibodies (*red*) and nuclear marker DAPI (*blue*). Ventral view, anterior up. Tp1:GFP expression in liver and pancreas of *jag1b* ^*−/−*^ mutants (**b**, representative sample, *n* > 15) is comparable to that in wild type (**a**), but is decreased in *jag2b* ^*−/−*^ mutants (**c**, representative of 14/18 samples). *jag1b* ^*−/−*^ *;jag2b* ^*−/−*^ double mutants (**d**, representative sample, *n* = 15) show a complete absence of Tp1:GFP expression in Prox1^+^ cells. **e** Bright-field microscopy of 4 dpf wild type (*top*) and *jag1b* ^*−/−*^ *;jag2b* ^*−/−*^ mutant (*bottom*), showing mutant with grossly normal development with the exception of heart and pronephric edema. Samples from three different *jag1b* ^*−/+*^ *;jag2b* ^*−/+*^ in-cross clutches. *Scale bars* 50 μM (**a**–**d**), and 500 μM **e**

Homozygous *jag1b* ^*b1105*^ mutant 72 hours post fertilization (hpf) embryos (*n* > 15) show no obvious decrease in Tp1:GFP expression in both the liver and pancreas (Fig. 1b). However, *jag2b* ^*hu3425*^ homozygous mutants (*n* = 26) do exhibit a reduction in the number of Tp1:GFP expressing cells. 77% of *jag2b* ^*hu3425*^ homozygotes have a partial loss of Tp1:GFP^+^ cells in both the liver and exocrine pancreas (Fig. 1c). The remaining embryos have 2, 1, or no Tp1:GFP expressing cells in the entire liver and exocrine pancreas (not shown), suggesting that Jag2b is the primary Notch ligand required for most Notch signaling during liver and pancreas development. The variable penetrance may be indicative of functional redundancy with other Notch ligands, as previously demonstrated by the more severe ductal defects in *jag1b* and *jag2b* knockdown studies^34^. However, Notch activity was not assessed in that knockdown study. We find that double homozygous embryos (*jag1b* ^*−/−*^ *;jag2b* ^*−/−*^) can survive past 72 hpf, when differentiation markers for pancreas acinar cells and hepatocytes are prominent. Bright field microscopy of double mutants indicates heart and pronephric edema to be the most obvious defects, with no apparent developmental delay (Fig. 1e). We find that all *jag1b* ^*−/−*^ *;jag2b* ^*−/−*^ embryos (*n* = 15 Fig. 1d) lack Tp1:GFP expression in the entire Prox1^+^ exocrine pancreas, while GFP can still be detected in the endocrine pancreas and the vasculature associated with the pancreas (Fig. 1a, Supplementary Fig. 3c). Loss of Notch activity is also observed in the Prox1^+^ liver domain, where approximately 33% of *jag1b* ^*−/−*^;*jag2b* ^*−/−*^ mutants also completely lack Tp1:GFP cells, while the remaining have only 1 or 2 Tp1:GFP^+^ cells (Supplementary Fig. 4a, b *n* = 15). These results indicate that Jag1b and Jag2b can be responsible for all detectable canonical Notch activity in the exocrine pancreas and liver. With a complete lack of canonical Notch signaling in *jag1b* ^*−/−*^ *;jag2b* ^*−/−*^ mutants, we have a more definitive model to investigate the specific role of ligand-induced canonical Notch signaling in liver and pancreas development.

### Intrahepatopancreas duct lineage specification requires Jagged

**Fig. 2:**
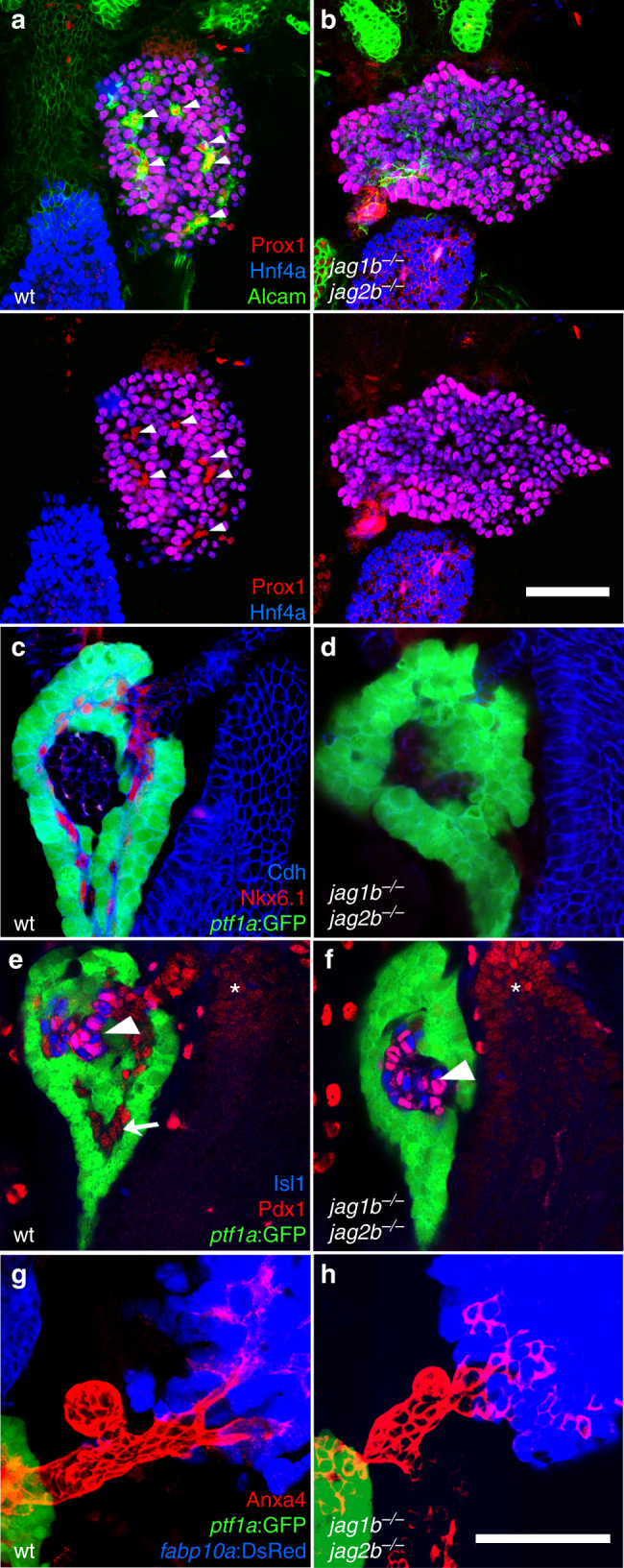
Jag1b and Jag2b are required for intrahepatopancreatic duct lineage specification. **a**–**h** Whole organ immunofluorescence expression analysis of the 72 hpf foregut endoderm of wild type and *jag1b* ^*−/−*^ *;jag2b* ^*−/−*^ mutant embryos. (**a**, **b** Alcam channel removed in *bottom panels*). Intrahepatic duct cells marked by high Alcama levels (**a**, *green*), and by Prox1 (*red*) but not Hnf4a expression (*blue*, **a**), are found in wild type liver (*arrowheads*, **a**). These duct cells are not found in the *jag1b* ^*−/−*^ *;jag2b* ^*−/−*^ mutant liver (**b**, representative sample, *n* = 13), which is comprised entirely of hepatocytes expressing both Prox1 and Hnf4a. **c**–**f** Intrapancreatic ductal cells expressing Nkx6.1 (*red*, **c**, **d**) or Pdx1 (*red*, *arrow*, **e**, **f**) are present in wild type but not in *jag1b* ^*−/−*^ *;jag2b* ^*−/−*^ mutants. Pdx1 expression in Islet1^+^ cells (*blue*, *arrowheads*) and the duodenum (*asterisk*) is comparable to wild-type siblings, as is expression of *ptf1a*:GFP^+^ (*green*) acinar cells **c**–**f** and Anxa4^+^ (*red*) extrahepatopancreatic duct cells (*red*, **g**, **h**). **g**, **h** Anxa4^+^ (*red*) extrahepatopancreatic ducts can be found joining the *ptf1a*:GFP^+^ (*green*) pancreatic acinar cells to the *fabp10a*:DsRed^+^ hepatocytes (*blue*). Representative samples from three different *jag1b* ^*−/+*^ *;jag2b* ^*−/+*^ in-cross clutches. *Scale bars*, 50 μM

To investigate the effect of canonical Notch signaling loss on the entire hepatopancreatic ductal system, we analyzed early markers distinguishing ducts from other pancreas and liver cell types. One of the earliest distinguishing features of the liver duct lineage is the lack of expression of the hepatocyte marker Hnf4a in Prox1 positive cells. As the liver matures, the hepatic duct cells maintain a high level of Alcama expression, whereas hepatocytes exhibit reduced or absent Alcama expression (Fig. 2a, Supplementary Fig. 4c). Whole-organ confocal microscopy revealed *jag1b* ^*−/−*^ *;jag2b* ^*−/−*^ livers with Hnf4a expressed in all Prox1^+^ cells, suggesting a complete lack of duct lineage. Consistently, high-level Alcama expression was almost completely lost (Fig. 2b), with the remaining expression associated with Prox1^+^ /Hnf4a^+^ cells (Supplementary Fig. 4d). Cleaved Caspase3, an apoptosis marker, was not observed in the double mutant liver (Supplementary Fig. 5a–d) or pancreas (not shown), suggesting the lack of duct cells is not due to cell death. These results indicate that Jag1b and Jag2b are essential for intrahepatic duct lineage specification.

During early ventral pancreas development in both mouse and zebrafish, *ptf1a* is initially co-expressed with *nkx6*.*1* and *pdx1*. As the duct and acinar lineages of the exocrine pancreas become specified, *ptf1a* expression becomes restricted to acinar cells while *nkx6*.*1* and *pdx1* become restricted to duct cells^45, 46^. By 72 hpf, *nkx6*.*1* and *pdx1* are only expressed in duct cells that have very low or undetectable *ptf1a* expression. In *jag1b* ^*−/−*^ *;jag2b* ^*−/−*^ embryos at 72 hpf, Nkx6.1 and Pdx1 are undetectable in the exocrine pancreas of all double mutants, whereas the pattern of *ptf1a*:GFP expression appears more contiguous throughout the exocrine pancreas (Fig. 2d, f). However, Pdx1 expression in the Islet1^+^ endocrine pancreas is not lost in the double mutants, indicating that Pdx1 loss is specific to the exocrine pancreas. These results indicate that Jag1b and Jag2b are essential for the lineage specification of the intrapancreatic duct. In *jag1b* ^*−/−*^ *;jag2b* ^*−/−*^ embryos, expression of Anxa4 in extrahepatopancreatic ducts^41, 47^ remains (EHPD; Fig. 2h), while the elevated expression normally observed in the IHPD cells is lost. This finding indicates that the EHPD can still form in *jag1b* ^*−/−*^ *;jag2b* ^*−/−*^ embryos, whereas the duct cells within the liver or pancreas are absent. This regionalized defect is consistent with detection of Notch activity in the IHPDs but not the EHPDs.

### Early and late roles of Notch in duct and endocrine lineages

**Fig. 3:**
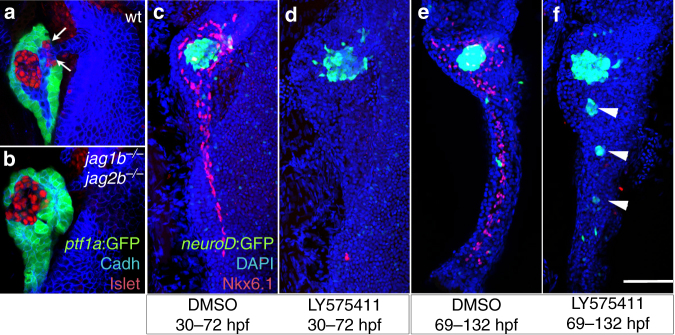
Distinct early and late roles of Notch signaling in regulating duct and endocrine lineages. **a**, **b** Pancreas of 60 hpf *jag1b* ^*−/−*^ *;jag2b* ^*−/−*^ and wt sibling embryos. Neogenic endocrine cells expressing Islet1 (*red*) are normally found outside the principal islet at the base of the wild type pancreas (*arrows*, **a**). These neogenic endocrine cells are not present in the 60hpf *jag1b* ^*−/−*^ *;jag2b* ^*−/−*^ mutant pancreas (**b**, representative sample, *n* = 18, pooled from three different *jag1b* ^*−/+*^ *;jag2b* ^*−/+*^ in-cross clutches), which is entirely *ptf1a*:GFP^+^ (*green*) with the exception of the principal islet. **c**, **d** Pancreas of 5 dpf-treated embryos. Early inhibition of Notch signaling with gamma secretase inhibitor LY575411 treatment (**d**, *n* = 9 and **c**, *n* = 5. Samples from 2 different experiments) at 30–72 hpf leads to loss of Nkx6.1^+^ pancreatic duct (*red*) without excess *neuroD*:GFP^+^ (*green*) endocrine cells. **e**, **f** In contrast, late LY575411 treatment (**f**, *n* = 9, and *n* = 6 for control, **e** Samples pooled from 2 different experiments) also leads to loss of nkx6.1^+^ pancreatic duct (*red*) that is coupled with excess *neuroD*:GFP^+^ (*green*) endocrine cells (*arrowheads*, **f**). *Scale bar*, 50 μM

Different models of Notch inhibition can result in a loss of duct cells that is associated with either excessive or reduced endocrine cell differentiation^20, 39, 48, 49^. In the *jag1b* ^*−/−*^ *;jag2b* ^*−/−*^ mutants, all pancreatic endocrine cells (Islet1^+^ or *neuroD*:GFP^+^) were found closely associated with the principal islet (100% *n* = 18, Fig. 3a, b), suggesting the absence of endocrine neogenesis. Consistently, the total number of pancreatic endocrine cells in the double mutants (87 (±6) Islet^+^ cells, *n* = 8) is significantly lower than in wild type at 89 hpf (113 (±23) Islet^+^ cells, *n* = 10). Our analysis does not show a consistent change in the size of the acinar pancreas in double mutants (see Figs. 2c-f and 3a,b).

To formally test whether the contrasting endocrine phenotypes among different Notch loss-of-function models reflect temporally distinct functions of Notch signaling during pancreas development, we blocked Notch signaling at different stages of pancreas development using a gamma secretase inhibitor (GSI), LY411575^50^. While the function of gamma secretase has been implicated in multiple signaling pathways^51^, chemical inhibitors of gamma secretase, including LY411575, has become an accepted approach to effectively block the Notch pathway in multiple animal models and organ systems. Importantly, we find that consistent with *jag1b* ^*−/−*^ *;jag2b* ^*−/−*^ embryos, LY411575 treatment starting prior to specification of the ventral pancreas leads to a total absence of Nkx6.1 positive pancreas duct cells at 5 days post fertilization (dpf) (Fig. 3d). All *neuroD*:GFP^+^ pancreas endocrine cells were found closely localized to the principal islet and not in the tail of the pancreas, suggesting a lack of excessive endocrine mis-differentiation. Although later treatment with LY411575 also leads to a loss of Nkx6.1 expressing duct cells, this defect is coincident with excessive *neuroD*:GFP^+^ endocrine cells in the tail of the pancreas (Fig. 3f). Together, our findings support a model whereby Notch signaling function is initially required for pancreatic duct lineage specification and indirectly for neogenic endocrine lineages, but is later required for maintenance of the ductal lineage via repression of endocrine fate. This model reconciles the contrasting data on the effect of Notch loss-of-function during pancreas endocrine and duct development. However, the possibility of the GSI treatment affecting other genetic pathways cannot be excluded. Further, in addition to temporal distinct roles for Notch signaling, spatial and ligands/receptors specific functions may also contribute to pancreas and liver development.

### Acinar pancreas and hepatocyte lineages do not require IHPD cells

**Fig. 4:**
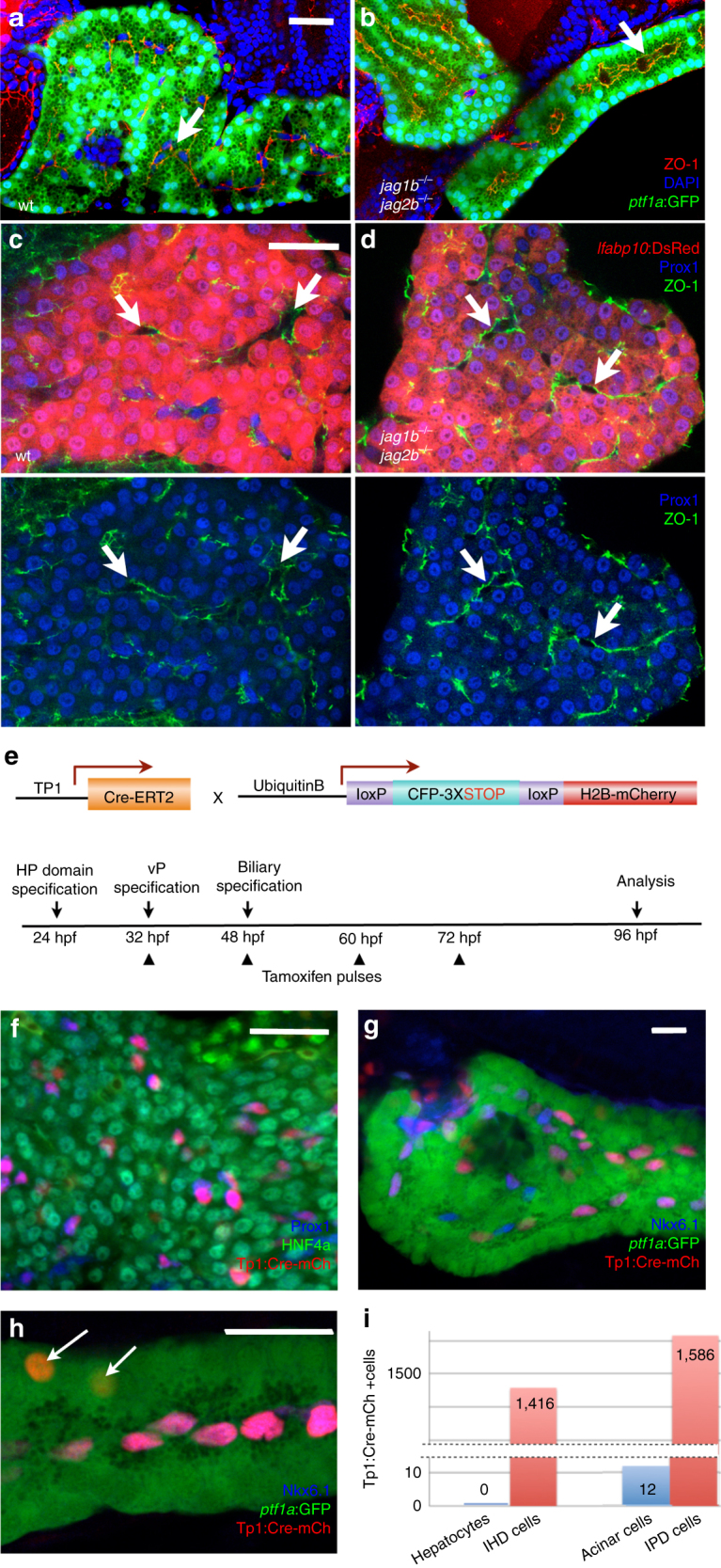
Intrahepatopancreatic ducts do not contribute significantly to the developing acinar and hepatocyte lineages. **a**–**d** Immunofluorescence analysis of the pancreas (**a**, **b**) and liver (**c**, **d**) in 5 dpf wild type (**a**, **c**) and *jag1b* ^*−/−*^ *;jag2b* ^*−/−*^ mutant embryos (**b**, **d**, *bottom* panels with *lfabp10:*DsRed channel removed). Despite the absence of ductal cells in *jag1b* ^*−/−*^ *;jag2b* ^*−/−*^ mutants **b**, **d**, lumen formation (*arrows*) and apical localization of ZO-1 (*red* in **b**, *green* in **d**) remain comparable to wild type siblings (**a**, **c**). Normal differentiation indicated by zymogen granule accumulation in the *ptf1a*:GFP^+^ pancreatic acinar cells (*green*) and *lfabp*-DsRed expression (*red*) in hepatocytes. **e** Schematic of approach to Notch activity lineage tracing. **f**–**i** Notch active labeled H2B-mCherry^+^ cells (*red*) in the liver are all Prox1^+^ (*blue*) and Hnf4a^−^ (*green*) (*n* = 12 embryos, 1,416 labeled cells counted from two different experiments (**f**), *P* < 0.0001). **g**, **h** In the pancreas, nearly all H2B-mCherry^+^ cells (*red*) co-express Nkx6.1 (*blue*) and are *ptf1a*:GFP^−^ (*green*) (*n* = 24 embryos, 1,586 labeled cells counted from 3 different experiments, *P* < 0.0001) **g** Only a few H2B-mCherry^+^ cells that lack Nkx6.1 but are *ptf1a*:GFP^+^ were observed (*arrows*, **h**. *n* = 4/24 embryos, 12 cells total). **i** Graph summarizing numbers of Notch active labeled H2B-mCherry^+^ cells found in hepatocytes versus intrahepatic duct (IHD) cells and acinar pancreas versus intrapancreatic duct (IPD) cells. Representative samples; **a**–**d**, *n* = 4 each. *Scale bars* 25 μM

*jag1b* ^*−/−*^ *;jag2b* ^*−/−*^ embryos provide a unique opportunity to examine the consequence of complete absence of canonical Notch signaling and duct cells during liver and pancreas organogenesis. Despite the mechanical pressure on the developing liver and pancreas by the adjacent heart and kidney edema found in these embryos, some double mutants do have livers and pancreata that are relatively normal in size and shape (Figs. 1–3). At 5 dpf, similar to the wild type pancreas, the double mutant acinar cells are polarized, as indicated by apical localization of ZO-1 (Figs. 4a, b) and zymogen granules. The formation of acinar lumens is obvious and can appear cystic in the double-mutant pancreas despite a complete lack of duct cells, providing evidence that duct cells are non-essential for exocrine pancreas lumen formation. In the *jag1b* ^*−/−*^ *;jag2b* ^*−/−*^ liver, ZO-1 is still detectable at the apical side of hepatocytes where lumens are apparent, despite also lacking duct cells (Figs. 4c, d). *fabp10a:DsRed* reporter^47^ expression can be found in all Prox1-expressing cells in the double mutant liver at 5 dpf, demonstrating broad hepatocyte differentiation (Fig. 4d). Together, these results lend functional evidence suggesting that intrahepatopancreatic ducts are not essential for the development of acinar and hepatocyte compartments of these organs.

Our discovery that Jagged/Notch signaling is the induction event for IHPD lineage specification suggests that tracking Notch active cells may be most reliable for assessing lineage contribution from duct cells. Further, the appearance of Tp1:GFP prior to the resolution of Nkx6.1, Pdx1, and Prox1 expression to the pancreatic ducts implicates Notch activity as the earliest known specific marker of the duct lineage. To determine whether Notch active cells contribute to the developing acinar and hepatocyte lineages, we lineage traced canonical Notch active cells using a transgenic line with tamoxifen inducible Cre driven by the Tp1 promoter (Tp1:CreER^T2^)^52^, in combination with a Cre reporter line, *ubb:lox-CFP-STOP-lox-H2b-mCherry* (Fig. 4e). Previous zebrafish pancreas development studies found a few Tp1:CreER^T2+^ cells contributing to the *ptf1a*:GFP^+^ acinar compartment with tamoxifen added at either 12 or 56 hpf^53^. To initiate lineage tracing of Tp1^+^ cells at the earliest stages of liver and pancreas development, we induced Tp1:CreER^T2^ activity starting at 30 hpf H2B-mCherry marked an average of 79% of Prox1^+^, Hnf4a-negative duct cells (Fig. 4g; total 1,416 cells, 12 embryos) in the liver and 75% of Nkx6.1^+^ and *ptf1a*:GFP or Elastase negative duct cells (Fig. 4f; total 1,586 cells, 24 embryos) in the pancreas. In some individual samples, over 90% of the intrahepatic ducts were labeled, demonstrating the effectiveness of this lineage tracing approach. We observed no labeling of hepatocytes (Hnf4a^+^) and very few of acinar cells (*ptf1a*:GFP^+^ or *elastase* ^+^, and Nkx6.1 negative). Of 24 embryos, 20 had no labeling of acinar cells, and 4 had a total of only 12 acinar cells labeled, versus the 2,578 labeled intrapancreatic duct cells in these same embryos. Collectively, our lineage tracing studies suggest that Notch active cells do not contribute to the hepatocyte lineage and potentially contribute very few cells to the acinar lineage during organogenesis.

### Endoderm Jagged induces Notch activity and duct lineage

**Fig. 5:**
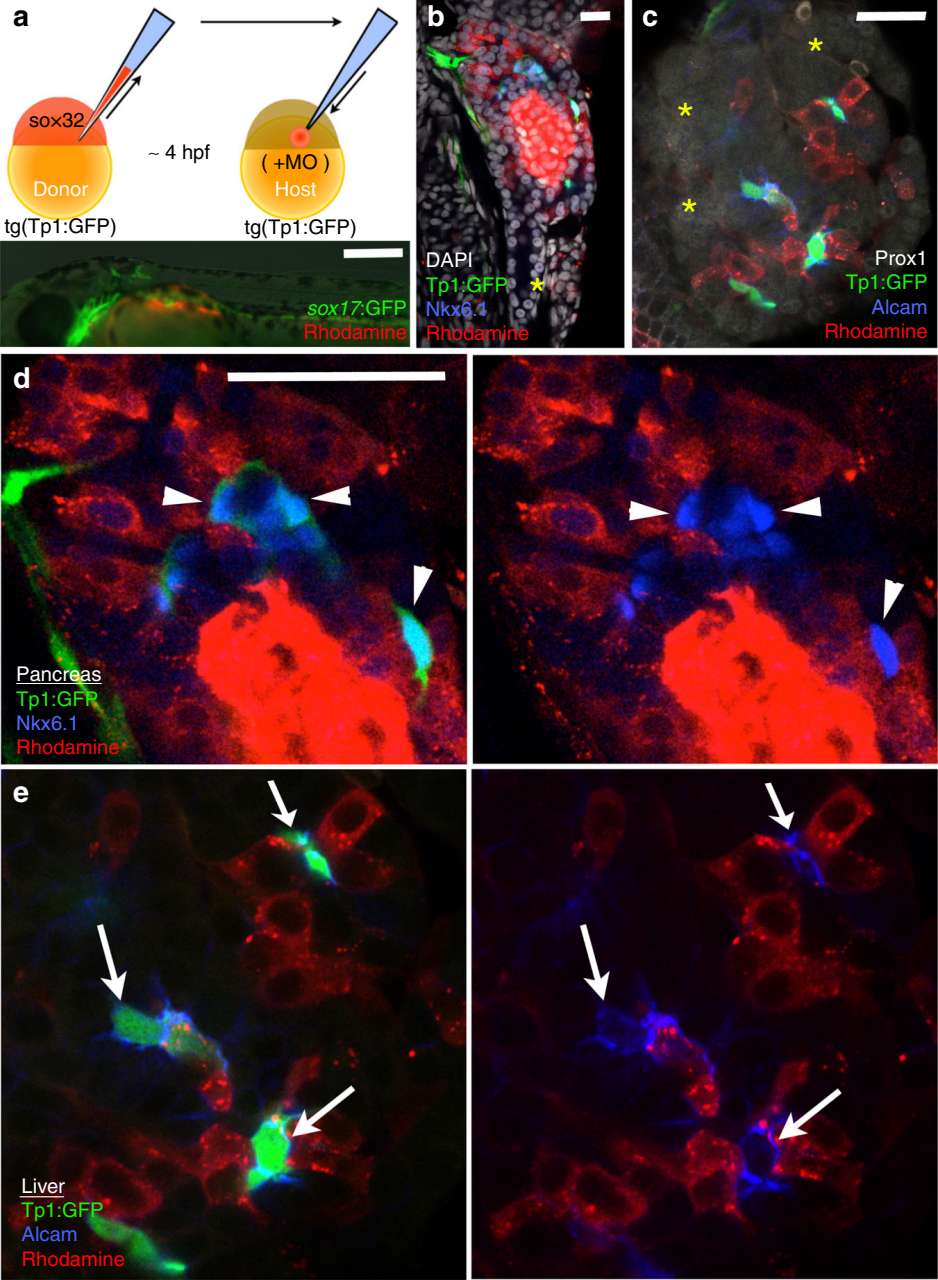
Endoderm expression of Jagged induces Notch activity and lineage specification. **a** Schematic of transplant approach to generate endoderm chimeras. At the one-cell stage, wild type *Tp1:GFP* donor embryos were injected with rhodamine dextran for lineage tracing and *sox32* mRNA for conversion to endoderm. At late blastula stage, donor cells were transplanted into *Tp1:GFP* host embryos that had been injected with antisense MO’s to knockdown Jag1a and Jag1b. (**a**, *bottom panel*) Example of endoderm chimeric embryo demonstrating rhodamine dextran labeled donor cells (*red*) localized primarily to the *sox17*:GFP^+^ (*green*) host gut endoderm region. **b**, **c** Immunofluorescence analysis of chimeric host pancreas (**b**) and liver (**c**) generated by the approach described in **a** demonstrating a rescue of Notch signaling in Jag1b/Jag2b compromised embryos by endoderm cells with wild type expression of Jag1b/Jag2b. Tp1:GFP^+^ cells (*green*) are only observed in regions of these organs with wild type donor cells (*red*) but not in regions without donor cells (*yellow asterisks*), (**d**, **e**) (representative samples, *n* = 10 embryos. Samples pooled from 2 experiments). Rescued Tp1:GFP^+^ cells found without rhodamine dextran (*red*, *white arrows* and *arrowhead*), are always directly associated with rhodamine dextran + cells. All rescued Tp1:GFP + host cells are Nkx6.1^+^ (*blue*) in the pancreas (**d**) and Alcama^+^ (*blue*) in the liver (**e**), indicating that they are of the duct lineage. *Scale bars* 250 μM **a**, and 25 μM (**b**–**e**)

Conditional inactivation of Jag1 from the portal vein mesenchyme led to liver duct tubulogenesis defects, but did not prevent the expression of Hes1, a marker of Notch signaling, or early liver duct markers, suggesting that Jag1 expression from the liver vasculature is not essential for liver Notch signaling or duct lineage specification^33^. Consistently, liver duct specification is also not lost in zebrafish *npasl4* mutants, which fail to develop the vasculature (Supplementary Fig. 6)^34, 54, 55^. It is not known whether endoderm sources of Jagged ligands are required to directly activate Notch signaling and induce IHPD specification. Jag1 was shown to be expressed endoderm-derived cells in the embryonic mouse liver and pancreas, and conditional loss of *Jag1* from the pancreatic endoderm using Pdx1-Cre leads to a postnatal reduction and malformation of pancreatic ducts^33, 48, 56^. These findings suggest that Notch signaling in these organs may be directly induced by Jagged signaling from adjacent endoderm-derived cells. To functionally test whether normal Jag expression in endoderm-derived cells of the liver and pancreas is sufficient for Notch activation and duct lineage specification, we analyzed embryos chimeric for *jagged* expression in the endoderm using a standard transplantation approach. We injected *sox32* mRNA to induce endoderm fate and a rhodamine dextran dye to fluorescently label the entire donor embryo. These labeled endoderm donor cells were then transplanted into unlabeled host embryos with *jag1b* and *jag2b* knocked down by morpholinos^57^ (Fig. 5a). The use of mutants was impractical for these transplantation experiments, as only 1/16 offspring are expected to be double homozygous for *jag1b* and *jag2b* from a double heterozygote incross. To assess canonical Notch activity, Tp1:GFP was used for both donor and host embryos. In embryos in which the pancreas and liver are chimeric (*n* = 10), there are no Tp1:GFP expressing cells found in regions of these organs that lack rhodamine dextran labeled wild type donor cells. However, Tp1:GFP^+^ cells were only found in regions of the organs with rhodamine dextran labeled wild type donor cells. Importantly, Tp1:GFP is observed in both donor (rhodamine^+^) and morphant host cells (rhodamine-) that are directly associated with the wild type donor cells (Figs. 5b, c). These Tp1:GFP cells are Nkx6.1^+^ in the pancreas and express high levels of Alcama in the liver, suggesting that they are indeed ductal cells. These findings suggest that Jagged ligands from endoderm derived cells in the liver and pancreas can directly induce Notch activity and IHPD lineage specification in adjacent cells.

## Discussion

**Fig. 6:**
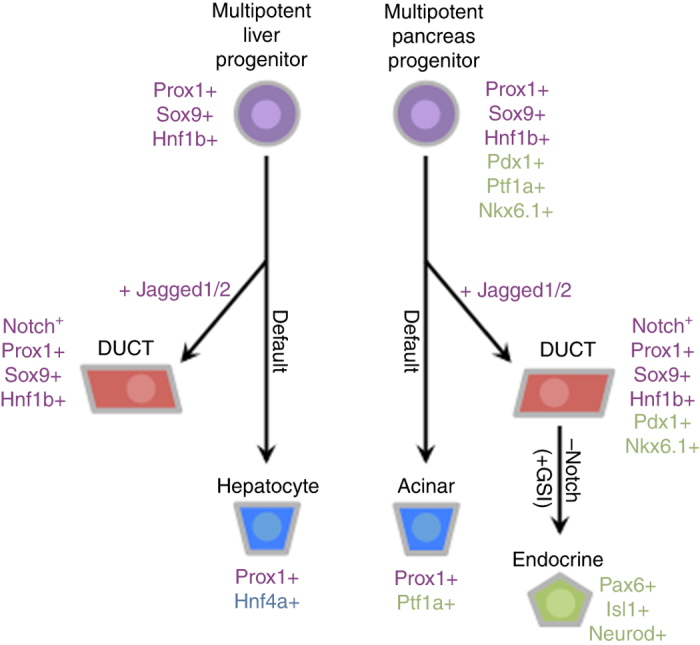
Model diagram depicting common role for Jagged1b and Jagged2b in lineage specification of liver and pancreas ducts in zebrafish. During organogenesis, multipotent liver and pancreas progenitors, by default, give rise to hepatocytes and acinar cells, respectively. Jagged/Notch signaling is required in both organs for induction of duct lineage specification from these multipotent precursors. Once Notch signaling is activated in a cell, it contributes exclusively to the duct lineage. In the pancreas, loss of Notch signaling via gamma secretase inhibitors (GSI) from lineage specified duct cells could lead to their mis-differentiation into endocrine cells. Factors listed in purple demonstrate genetic similarities of cell types between the pancreas and liver. In *green* and *blue* are factors specific to the pancreas and liver, respectively

The investigation presented here addresses several fundamental outstanding questions regarding intrahepatopancreatic duct specification, function, and disease. The unique ability to generate *jag1b/2b* compound homozygous embryos that are viable throughout organogenesis was critical for these studies. Our mutant analysis demonstrated that just two ligands, Jag1b and Jag2b, are responsible for all detectable canonical Notch signaling and duct lineage specification in both the exocrine pancreas and liver of zebrafish (summarized in Fig. 6). Notch signaling has been linked to cell cycle due to the pancreatic hypoplasia observed with loss of Notch associated factors Rbpjk, Hes1, and Sox9^58^. However, the lack of any detectable canonical Notch signaling and the normal pancreas size in *jag1b* ^*−/−*^ *;jag2b* ^*−/−*^ embryos suggest that the function of these nuclear factors in early pancreas growth is not associated with ligand-dependent canonical Notch signaling. *jag1b* ^*−/−*^ *;jag2b* ^*−/−*^ embryos also helped distinguish the roles of Notch signaling in duct lineage specification versus maintenance. Loss of pancreatic duct cells in these double mutants is not coupled with excessive endocrine differentiation as observed with other Notch loss-of-function approaches. Differences in stage and severity of Notch signaling loss may explain the phenotypic differences found. We conclude that during pancreas development, Notch signaling function is initially required for duct lineage specification and indirectly for neogenic endocrine lineages, and is later required for repression endocrine fate to maintain the ductal lineage (summarized in Fig. 6).

With a complete lack of duct lineage specification in double mutants, it was surprising to find that the developmental specification and morphogenesis of the acinar and hepatocyte compartments were not overtly affected. This finding was especially unexpected because the IHPDs are thought to play a central role in organogenesis, contributing progenitors to the development of the acinar and hepatocyte compartments^6, 13–15^. Our studies provide functional evidence using *jagged* mutants and more definitive lineage tracing data using the earliest duct specific reporter to reveal that ducts of the liver and pancreas are not essential for and do not contribute significant numbers of cells to the developing hepatocyte and acinar lineages. A profound understanding of liver and pancreas progenitor biology will shape our perspectives on regeneration and diseases associated with these organs such as cancer, diabetes, and Alagille syndrome.

In the complete absence of Jagged/Notch signaling in double mutant embryos, we posit that cells that would normally contribute to intra hepatopancreatic ducts are mis-specified to adopt acinar and hepatocyte fates. Because Jagged expressing cells function to induce Notch signaling and the duct lineage, and no duct specific genes are activated in these mutants, formal lineage tracing experiments to determine the lineage derivatives of presumptive ducts are currently not possible. However, it has been shown that ectopic Notch signaling by misexpression of the Notch intracellular domain can induce duct lineage markers in acinar cells and hepatocytes, suggesting that Notch signaling can promote ductal fates at the expense of hepatocyte and acinar fates^22, 23^. Together with our loss of function studies, we favor a model in which Notch functions during early pancreas and liver development to redirect acinar and hepatocyte precursors towards adopting duct fate instead (summarized in Fig. 6). This model argues that acinar and hepatocyte lineages are default in the absence of Notch signaling.

Based on mouse studies of Jag1 function, the prevailing model suggests that liver duct paucity in ALGS is caused by liver duct tubulogenesis defects that are due to insufficient JAG1 signals from non-endoderm derived cells^33^. However, the findings presented here suggest that ductal paucity in ALGS might also be due to compromised duct lineage specification and to decreased Jagged signaling from endoderm-derived cells in the liver and pancreas. *Jag1* heterozygous mice exhibit a liver duct paucity defect only when they are extensively back-crossed into a C57BL/6 J background^59^, suggesting that other genes are involved. Consistently, *Jag1* and *Notch2* double heterozygous mice do exhibit biliary paucity^60^. However, this defect was attributed to a duct morphogenesis failure rather than a failure in cell fate specification^32^. Although the contrast between our finding of a duct lineage specification defect due to loss of both Jag1b and Jag2b Notch ligands and the reported duct morphogenesis defect with loss of Jag1 is likely a result of differential levels or timing of Jagged loss-of-function, species-specific differences cannot be ruled out. Future knockout of both Jag1 and Jag2 from mouse hepatopancreatic progenitors could reveal a conserved role for Jagged ligands in duct lineage specification in mammals. If compromised duct lineage specification is indeed a contributing factor to ductal paucity in ALGS patients, we will need to rethink the therapeutic strategies for this pathology and consider a regenerative approach to replace lost duct cells in this disease.

## Methods

### Zebrafish strains

Zebrafish were raised and maintained under standard conditions. The following mutants and transgenic lines were used: *jag1b* ^*b1105*^ ^38^; *jag2b* ^*hu3425*^, *npasl4* ^*s5*^ (*cloche*)^55^, *Tg(Tp1bglob:eGFP)* ^*um14*^ ^39^, abbreviated as *Tg(Tp1:GFP)*; *Tg(ptf1a:GFP)* ^*jh1*^ ^61^; *Tg(lfabp10:DsRed*, *elaA:EGFP)* ^*gz12*^ ^62, 63^; *Tg(sox17:GFP)* ^*s870*^ ^64^, *Tg(neuroD:EGFP)* ^*nl1*^ ^65^; Tg(Tp1:CreER^T2^)^7^. Tg(*UBB:loxP-CFP-STOP-Terminator-loxP-hmgb1-mCherry)* ^*jh*^ ^66^ transgenic fish were made from a transgene modified from the *β-actin:loxP-CFP-loxP-hmgb1-mCherry* construct. The *β-actin* promoter was replaced with promoter/enhancer sequence from *ubiquitinB* (*ubb*)^66^. To minimize read through, three stop codons were added after the CFP open reading frame along with *Cauliflower mosaic virus* (*CaMV*) 35S terminator sequence^67^ from plasmid pCSKalTA4GI^68^ and two SV40 polyA signals. This Cre-responder construct was injected with Tol2-transposon RNA to generate mosaic F0 fish^69^. Multiple F1 lines were screened for ubiquitous blue fluorescence and Cre-dependent nuclear red fluorescence. One line (*jh63*) was selected for further use as it displayed a high level of fluorescent marker expression along with high sensitivity to Cre recombination.

### Immunohistochemistry

Mutant and wild-type siblings were anesthetized and washed for 1.5 min in PBS with .3% Triton X-100 for permeabilization, then fixed with 2% formaldehyde in 0.1 M PIPES, 1.0 mM MgSO_4_ and 2 mM EGTA overnight at 4 °C. The embryos were manually deyolked under a dissecting microscope and blocked for 1 hour in PBS with 4% BSA and 0.3% Triton X-100. Primary and secondary antibodies were incubated overnight. Washes were done after each step with 0.3% Triton X-100 in PBS for 3 h to remove excess antibodies. Antibodies used include mouse monoclonal anti-Nkx6.1 (1:20; Developmental Studies Hybridoma Bank (DSHB), Iowa), rabbit polyclonal anti-Prox1 (1:200; Millipore), goat polyclonal anti-Hnf4a (1:50, Santa Cruz), rabbit polyclonal anti-pan-Cadherin (1:5000; Sigma), mouse monoclonal anti-Islet1/2 (1:20; DSHB), mouse monoclonal 2F11 anti-Anxa4^41, 70^ (1:1000; gift from J. Lewis, Cancer Research UK), mouse monoclonal anti-Alcama (1:20; DSHB), mouse anti-ZO1 (1:200, Zymed), chick or rabbit polyclonal anti-GFP (1:300; Torrey Pines Biolabs), rabbit ﻿anti-cleaved Caspase-3 (1:50; Cell Signaling). Imaging was done with a Zeiss 710 confocal microscope with Zen9 software and images were processed with Adobe Photoshop CS3.

### Lineage tracing and cell counting

For CreER^T2^ induction, embryos were incubated in 20uM 4-Hydroxytamoxifen (4-OHT, T176, Sigma, St. Louis, MO) for 2 h pulses, beginning at 42 hpf every 12 h until 72 hpf 4-OHT was dissolved in 100% DMSO for a stock solution of 10 mM, and dissolved in embryo media. For quantification of lineage traced cells, embryos were stained for Hnf4a, Prox1, mCherry and DAPI for liver analysis; and Nkx6.1, mCherry and DAPI for pancreas, with either Elastase or *ptf1a*:eGFP, for pancreatic analysis. mCherry positive cells were manually counted using Zen9 software in confocal z-stack images of whole livers and pancreas. To quantify endocrine cell number in *jagged* mutants, offspring embryos from *jag1b* ^*−/+*^ *;jag2b* ^*−/+*^ parents were fixed at 87 hfp, stained for Pdx1, Nkx6.1 and DNA, and imaged using confocal microscopy. Total Islet^+^ cells inside and outside the primary Islet were manually counted from z-stack images, and individual samples thereafter genotyped as described above. Control and experimental samples were scored blinded when possible.

### Whole-mount fluorescent in situ hybridization

For whole-mount ISH, zebrafish embryos were fixed in 4% paraformaldehyde (PFA) and dehydrated in methanol. Rehydrated embryos were deyolked under a bright-field dissecting microscope, treated with 1.5 μg/ml proteinase K for 75 or 90 min for 56 and 72 hpf embryos, respectively, post-fixed with 4% PFA for 20 min, then washed four times in phosphate-buffered saline with 0.1% Tween-20 (PBST). The prepared embryos were then pre-hybridized (50% formamide, 5x saline sodium citrate (SSC) buffer, 100 μg/ml yeast RNA, 50 μg/ml heparin, 0.125% Tween-20; pH to 6.0 with citric acid) at 68.5 °C for four hours and then incubated overnight with *jag1b* and *jag2b* probes^38^. The following day, embryos were subjected to stringency washes in a formamide/SSC series, washed twice in PBST at room temperature, treated with 2% H_2_O_2_ in PBST for one hour, washed four times in TNT buffer (100 mM Tris-HCl, pH 7.5, 150 mM NaCl, 0.1% Tween-20), and blocked in 0.5% Perkin Elmer blocking reagent in TNT for four hours. After incubating overnight with an anti-DIG-horseradish peroxidase antibody (1:400; Roche) at 4 °C, the embryos were washed eight times in TNT, pre-treated with PE Amplification Diluent for 5 minutes, and then exposed to TSA Cyanine3 (1:60; Perkin Elmer) for one hour. The reaction was quenched by treatment with 2% H_2_O_2_ in TNT for one hour, and the embryos were washed in PBS and immunostained with a rabbit anti-GFP antibody (1:1000; Torrey Pines Biolabs) and an Alexa488-conjugated goat anti-rabbit secondary antibody (1:300; Molecular Probes). The DRAQ5 nuclear stain (1:500; BioStatus) was applied with the secondary antibody.

### Morpholino injection and endoderm transplantation

Translation blocking morpholinos for *jag1b* (5′-CTGAACTCCGTCGCAGAATCATGCC-3′) and *jag2b* (5′- TCCTGATACAATTCCACATGCCGCC-3′^34^ (Gene Tools) were combined and diluted in 0.2 M KCl and phenol red from a 8.5 mg/ml stock to 2 mg/ml (each) working concentration. One nanoliter was injected for a final amount of 2 nanograms each into the host embryos. Donors embryos were injected with 100 picograms of *sox32* mRNA,and rhodamine dextran at the one cell stage. At 4 hpf, both donor and host embryos were dechorionated in an agarose-coated petri dish. Donor cells were drawn into a glass needle (pulled from Borosilicate Glass Capillaries, World Precision Instruments, Inc.) using CellTram Air (Eppendorf). About 15 donor cells were then injected into one host embryo using CellTram Air.

### Chemical inhibitor treatment

Notch signaling was inhibited at various stages of pancreas development using a gamma-secretase inhibitor (GSI), LY411575^50^. Larvae were treated with 50 μM LY411575 (Sigma) in egg water with 1% DMSO^50^. Sibling larvae from the same clutch were treated with 1% DMSO in egg water as control. Embryos were treated from 28-132 hpf for early inhibition, and from 69 hpf-132 hpf for late inhibition.

### Statistical analysis

Calculations were made in Microsoft Excel. We report mean and standard deviation, the probability associated with the Student’s t test (with two-tailed distribution). *P* values were calculated by Student’s t-test for single comparisons of normally distributed data. *P*-value was obtained through comparison of data sets: the expected number of acinar or hepatocyte cells labeled verses the actual number of acinar or hepatocyte cells labeled. Expected number of acinar and hepatocytes were based on Cre efficiency multiplied by a conservative estimates of acinar:ductal cell ratio of 3:1^71^ and hepatocyte:ductal cell ratio of 6:1^40^. We used GraphPad online software to perform Student’s t test (with two-tail distribution). The two-tailed *P* values are less than 0.0001. By conventional criteria, this difference is considered to be extremely statistically significant.

### Data availability

The authors declare that all data supporting the findings of this study are available within the article and its Supplementary Information Files or from the corresponding author upon reasonable request.

## Electronic supplementary material

Supplementary information
