# Supplementary material for: Endoderm Jagged induces liver and pancreas duct lineage in zebrafish
Source: Nat Commun. 2017 Oct 3;8:769. doi: 10.1038/s41467-017-00666-6 (PMC5626745; doi:10.1038/s41467-017-00666-6)
Supplement: Supplementary file 1 — Supplementary information [file 41467_2017_666_MOESM1_ESM.pdf]

SI GUIDE

File Name: Supplementary Information
Description: Supplementary Figures 1-6.

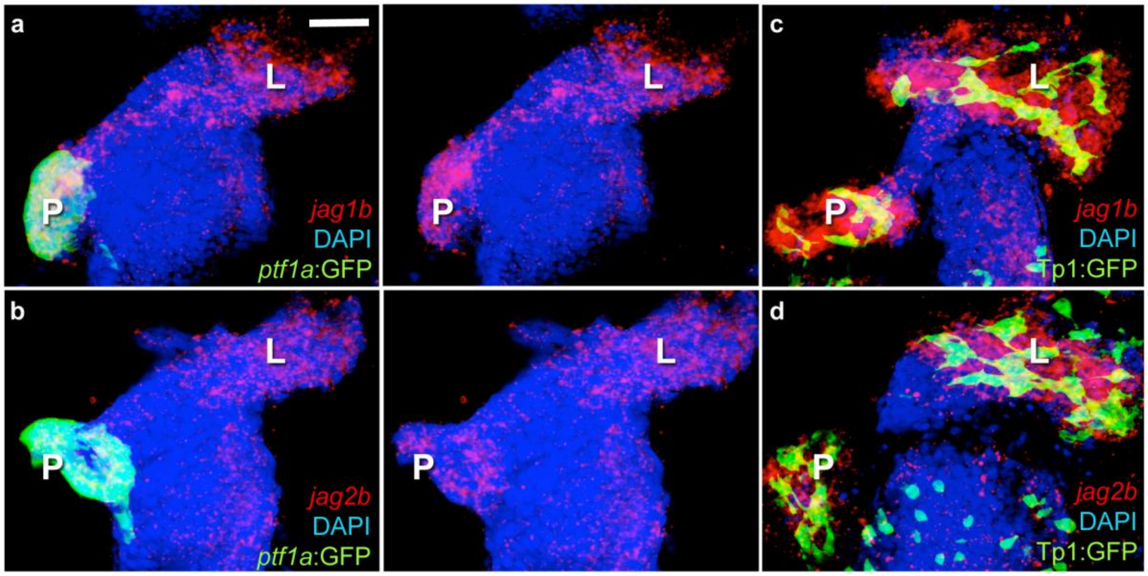

Supplementary Figure 1. *jag1b* and *jag2b* expression in the developing pancreas and liver.

Ventral view, 3D confocal rendering of whole mount fluorescent *in situ* hybridization detecting *jag1b* and *jag2b* expression in *ptf1a:GFP* (green, pancreas) 56 hpf embryo (a-b, panels to right with GFP channel removed) and *Tp1:GFP* (green) 72 hpf embryos (c-d). mRNA expression of *jag1b* (a, c) and *jag2b* (b, d) is prominent in both the liver (L) and pancreas (P). Representative samples, n= 4 each. Scale bars 50μM.

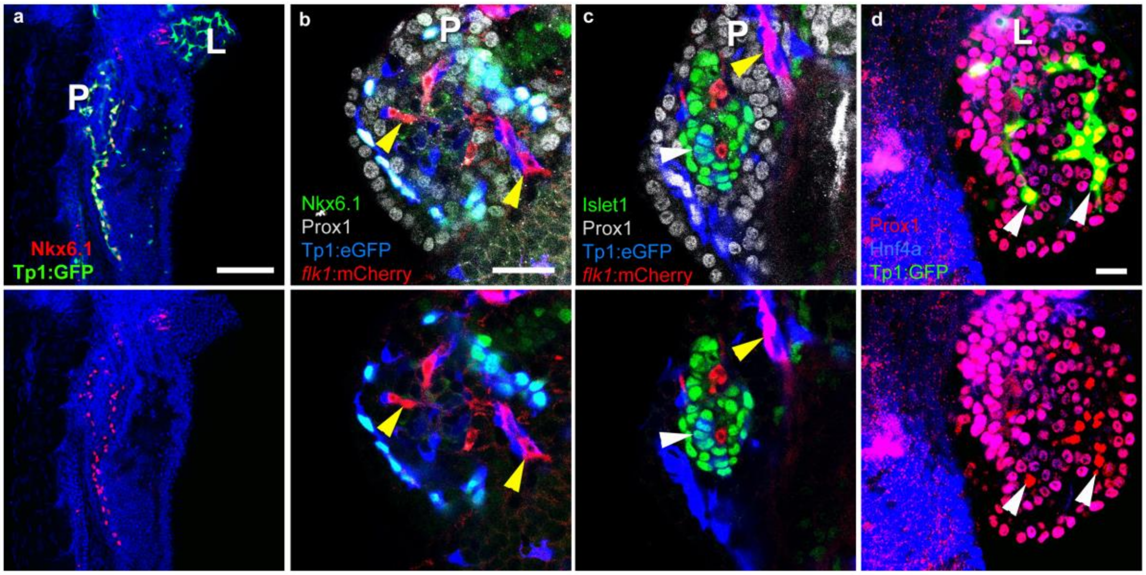

Supplementary Figure 2. Canonical Notch activity in liver and pancreas. (a-c) Immunofluorescence analysis showing all Nkx6.1+ cells in the pancreas (P) are Tp1:GFP+ (green, Notch active) (a). (**b-c**) Notch active, Tp1:GFP+ cells (blue) endothelial cells expressing *flk1*:mCherry (red) can also be found associated with the pancreas. These endothelial cells (magenta, yellow arrowheads) do not expression Prox1 (gray), Nkx6.1 (green, duct, b), or Islet1 (green, endocrine, c). However, a subset of Islet1+ endocrine cells of the pancreas can be Tp1:GFP+ (c, white arrowheads). **(d)** Prox1+ (red) cells in the liver that are Tp1:GFP+ (green) are always observed to not express Hnf4a (blue), indicative of intrahepatic duct cells (white arrowheads). Bottom panels with TP1:GFP (a, d) or DAPI (b,c) channels removed. Scale bars 100μM (a), and 25μM (b-d)

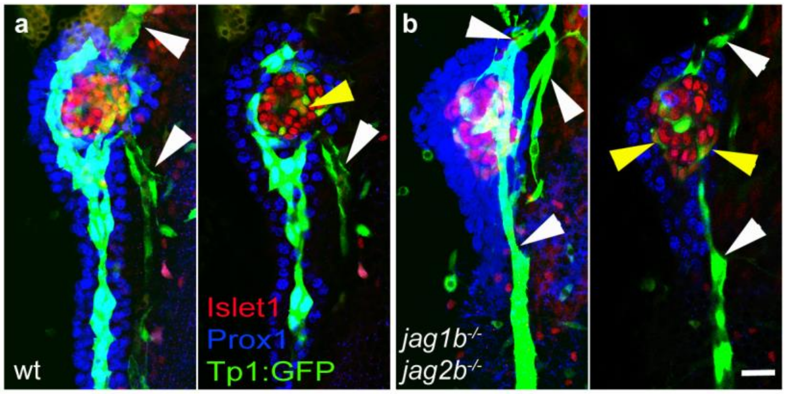

Supplementary Figure 3. Jag1b and Jag2b are not required for all Notch active endocrine or endothelial cells associated with pancreas. (**a-b,** Three dimensional renderings, single plane in panels to right) Pancreas of wild type (a, representative sample n=9) and *jag1b$^{-/-}$;jag2b$^{-/-}$* mutant (b, representative sample n=4) 72 hpf embryos showing loss of Tp1:GFP from all Prox1+ (blue) cells in mutants. Tp1:GFP expression can still be found in endothelial cells (white arrowheads) and Islet1+ endocrine cells (yellow arrowheads) Scale bar 25μM

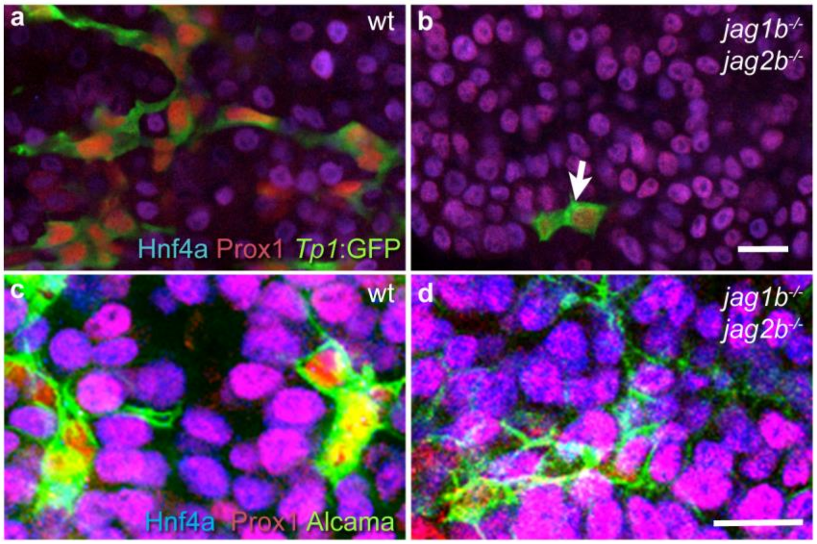

Supplementary Figure 4. **High magnification of *jag1b* and *jag2b* double mutant livers.** (**a-b**) Wild type (a) and *jag1b*$^{-/-}$;*jag2b*$^{-/-}$ mutant (b) livers labelled for Prox1 (red), Hnf4a (blue), and Tp1:GFP (green) reveals some double mutant embryos retain 1-2 intrahepatic duct cells (arrow, b, representative sample, 12/17 double mutants) (**c, d**) Enlargement from Figure 2a-b of wildtype (c) and *jag1b*$^{-/-}$;*jag2b*$^{-/-}$ livers (d) labeled for Prox1 (red), Hnf4a (blue), and Alcama (green). Low-level Alcama expression that persists in mutants is otherwise associated with cells expressing both Prox1 and Hnf4a. Scale bars, 25μM

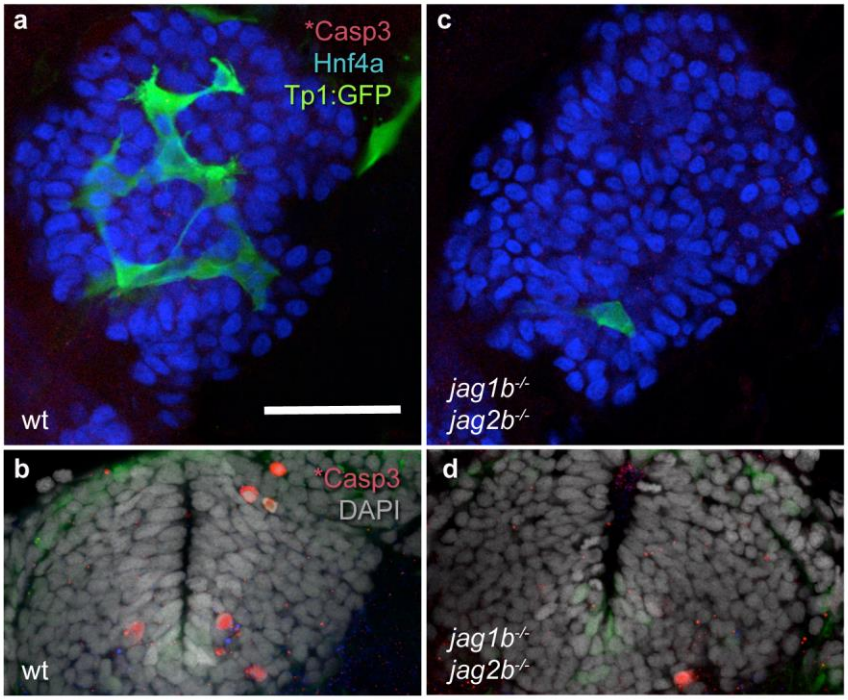

Supplementary figure 5. Apoptosis does not contribute to the lack of intrahepatic duct cells in *jag1b* and *jag2b* double homozygous mutants. (**a-d**) Whole mount immunofluorescent images of 54 hpf sibling (a,b) and double mutant (c,d) embryos labeled for cleaved Caspase3 (*Casp3) expression. Sibling (a) and double mutant (c) livers labeled for Hnf4a (blue) show no signs of apoptosis based on a lack of cleaved Caspase3 (red) expression or blebs of Tp1:GFP+ (green), whereas the ventral forebrains (b,d, with DAPI channel also shown) of the same embryos (from a,c) show normal apoptosis patterns as indicated by variable presence of cleaved Caspase3 (red). Scale bar 50μM. Representative samples, n= 4 siblings, 4 *jag1b*$^{-/-}$;*jag2b*$^{-/-}$

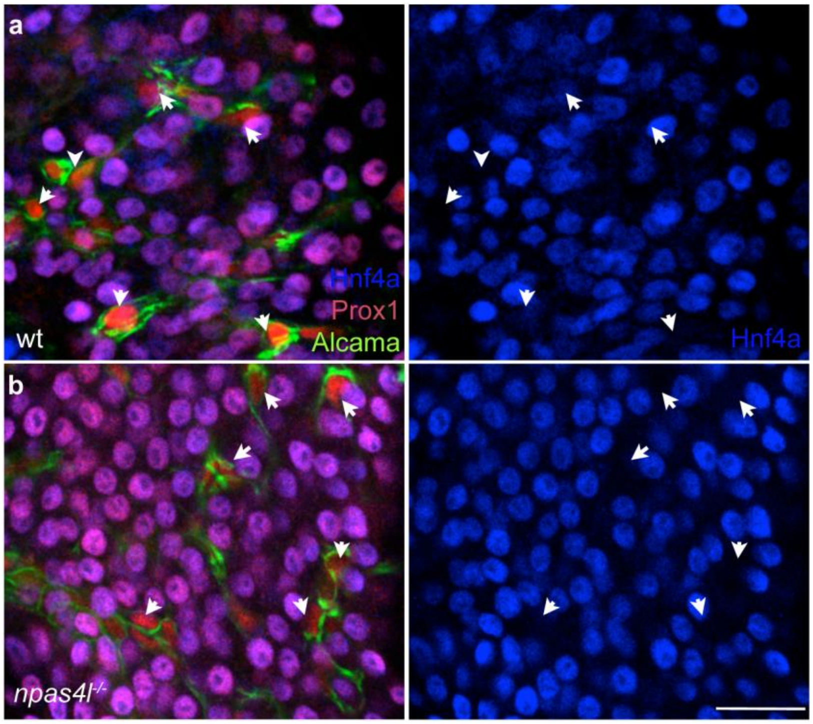

Supplementary figure 6. **Specification of intrahepatic duct lineage in the absence of the vasculature.** (**a-b**) Whole organ immunofluorescent analysis of 76 hpf wild type (a) and *npas4l* mutant (b) livers labelled for Prox1 (red), Hnf4a (blue), and Alcama (green). Green Alcama channel is removed in panels below a and b. Intrahepatic duct cells (arrows), expressing both Prox1 and high Alcama, but lacking Hnf4a, are present in both wild type and mutant livers. Scale bars 20μM
